# A Thermophilic Ionic Liquid-Tolerant Cellulase Cocktail for the Production of Cellulosic Biofuels

**DOI:** 10.1371/journal.pone.0037010

**Published:** 2012-05-23

**Authors:** Joshua I. Park, Eric J. Steen, Helcio Burd, Sophia S. Evans, Alyssa M. Redding-Johnson, Tanveer Batth, Peter I. Benke, Patrik D'haeseleer, Ning Sun, Kenneth L. Sale, Jay D. Keasling, Taek Soon Lee, Christopher J. Petzold, Aindrila Mukhopadhyay, Steven W. Singer, Blake A. Simmons, John M. Gladden

**Affiliations:** 1 Joint BioEnergy Institute (JBEI), Physical Biosciences Division, Lawrence Berkeley National Laboratory, Berkeley, California, United States of America; 2 Bioengineering & Biomass Science and Conversion Technology Department, Sandia National Laboratories, Livermore, California, United States of America; 3 Physical and Life Sciences Directorate, Lawrence Livermore National Laboratory, Livermore, California, United States of America; 4 Department of Geochemistry & Department of Ecology, Earth Sciences Division, Lawrence Berkeley National Laboratory, Berkeley, California, United States of America; 5 College of Chemistry, University of California, Berkeley, California, United States of America; Cinvestav, Mexico

## Abstract

Generation of biofuels from sugars in lignocellulosic biomass is a promising alternative to liquid fossil fuels, but efficient and inexpensive bioprocessing configurations must be developed to make this technology commercially viable. One of the major barriers to commercialization is the recalcitrance of plant cell wall polysaccharides to enzymatic hydrolysis. Biomass pretreatment with ionic liquids (ILs) enables efficient saccharification of biomass, but residual ILs inhibit both saccharification and microbial fuel production, requiring extensive washing after IL pretreatment. Pretreatment itself can also produce biomass-derived inhibitory compounds that reduce microbial fuel production. Therefore, there are multiple points in the process from biomass to biofuel production that must be interrogated and optimized to maximize fuel production. Here, we report the development of an IL-tolerant cellulase cocktail by combining thermophilic bacterial glycoside hydrolases produced by a mixed consortia with recombinant glycoside hydrolases. This enzymatic cocktail saccharifies IL-pretreated biomass at higher temperatures and in the presence of much higher IL concentrations than commercial fungal cocktails. Sugars obtained from saccharification of IL-pretreated switchgrass using this cocktail can be converted into biodiesel (fatty acid ethyl-esters or FAEEs) by a metabolically engineered strain of *E. coli*. During these studies, we found that this biodiesel-producing *E. coli* strain was sensitive to ILs and inhibitors released by saccharification. This cocktail will enable the development of novel biomass to biofuel bioprocessing configurations that may overcome some of the barriers to production of inexpensive cellulosic biofuels.

## Introduction

Growing worldwide energy demands and the threat of global warming has led nations to seek alternative sources of renewable energy derived from wind, solar, and biomass. The transportation sector relies mainly on liquid fuels, such as gasoline and diesel, because they are energy dense and fungible. Development of renewable liquid fuels, like bioethanol and advanced biofuels will reduce reliance on fossil fuels. Currently, bioethanol is produced in the United States mostly by hydrolysis and fermentation of corn starch. Yet, starch from corn ethanol may not be the ideal carbon source for fuel production in the long term [1]. Cellulosic biomass provides a more sustainable source of fermentable sugar and it is estimated that a billion tons are available annually in the US [2]. Roughly half of that biomass is composed of cellulose that, after hydrolysis to glucose, can be fermented into cellulosic biofuels [3], [4]. Some of the few cellulosic biofuel companies in operation the US, such as Poet (www.poet.com), extract glucose from biomass by physical and chemical pretreatment to reduce its recalcitrance followed by enzymatic saccharification to release glucose from plant cell wall polymers [5], [6], [7].

**Figure 1:**
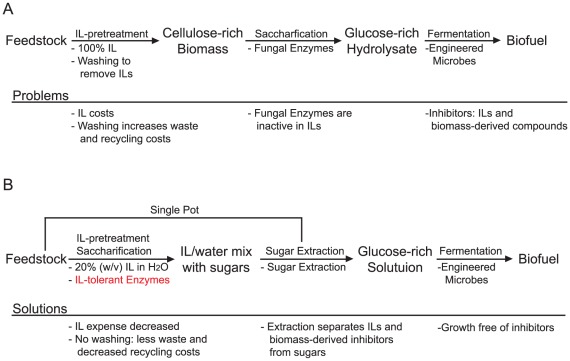
A flow diagram of two potential biomass-to-biofuel bioprocessing configurations that utilize IL-pretreatment. A) Diagrams a configuration based on methods currently established in the literature and lists some potential barriers to commercialization (Problems). B) This configuration combines IL-pretreatment and saccharification into a single pot and may overcome barriers outlined in A (as listed in the solutions section), but requires an IL-tolerant cellulase cocktail, such as JTherm.

Biomass recalcitrance is a difficult barrier to commercial deployment of cellulosic biofuels, yet there are a few promising pretreatments currently available or under development. For example, pretreatment with ionic liquids (ILs), such as 1-ethyl-3-methylimidazolium acetate ([C2mim][OAc]) dramatically reduces biomass recalcitrance and enhances the enzymatic hydrolysis of fermentable sugars [8], [9]. The bioprocessing configuration in Figure 1A outlines an IL-pretreatment scheme that reflects the current state of the art (based on a survey of the literature) and highlights some potential problems associated with this configuration. These problems include: 1) IL-pretreatment is frequently conducted using 100% IL at high temperatures (120–160°C) [8], [10]; 2) ILs are currently expensive so any viable bioprocessing scheme must include efficient IL recycling [11]; 3) this pretreatment configuration requires extensive washing of the biomass post-pretreatment to completely remove ILs, which can inhibit downstream saccharification and fermentation [12], [13], [14]. Washing has a negative impact in this scheme, increasing costs through energy-intensive evaporation or reverse osmosis recycling of ILs.

More recent studies have shown that lower IL concentrations (25–50% w/v) in water may also be effective in pretreating biomass, potentially reducing the amount of washing required prior to enzymatic saccharification [15]. Pretreament with these lower IL concentrations presents the possibility to explore alternate, potentially more inexpensive, bioprocessing configurations in which the washing step is removed. The configuration in Figure 1B outlines one potential IL/water-based scheme that combines IL-pretreatment and saccharification into a single pot, followed by direct extraction of sugars. Brennen et al. demonstrated that boronate complexes can extract up to 90% of sugars from an aqueous IL solution [16]. In this configuration, the boronate extraction method could be used to separate the sugars away from the ILs, eliminating the requirement for extensive washing. Boronate extraction may also separate sugars from any biomass-derived inhibitors, which have been associated with other types of pretreatment but have not been fully investigated in regards to IL-pretreatment [17]. Other methods to extract sugars, such as chromatography or membrane-based separation, could also potentially be used at this step.

The single-pot configuration requires biomass-deconstructing enzymes that are tolerant to concentrations of >20% IL. At these high IL concentrations, glycoside hydrolase enzymes in commercial biomass-degrading enzyme cocktails derived from filamentous fungi are inactive [12], [18]. However, glycoside hydolases have been isolated from thermophilic and halophilic microbes that tolerate up to 30% IL, suggesting that these enzymes may be good targets for the development of IL-tolerant cellulase cocktails [12], [18], [19], [20]. These highly stable enzymes will be of use in IL-pretreatment based bioprocessing platforms, and may also benefit platforms that utilize thermophilic fuel production hosts, such as *Clostridium thermocellum* or *Thermoanaerobacterium saccharolyticum*, for simultaneous saccharification and fermentation (SSF) [21], [22], [23]. In this study, we used thermoohilic enzymes to develop an IL-tolerant cellulase cocktail, called “JTherm”, which is compatible with the single-pot bioprocessing configuration outlined in Figure 1B. We took a hybrid approach where we combined native enzymes produced by a thermophilic bacterial community with recombinant thermostable enzymes. In addition, to validate the IL-pretreatment bioprocessing scheme in Figure 1A we assessed the impacts of hydrolyzates of IL-pretreated biomass produced by both JTherm and a commercial cocktail on fuel production in an *E. coli* strain engineered to produce FAEEs, a fuel equivalent to biodiesel.

## Results

### A thermophilic bacterial consortium with biomass degrading activity

**Figure 2:**
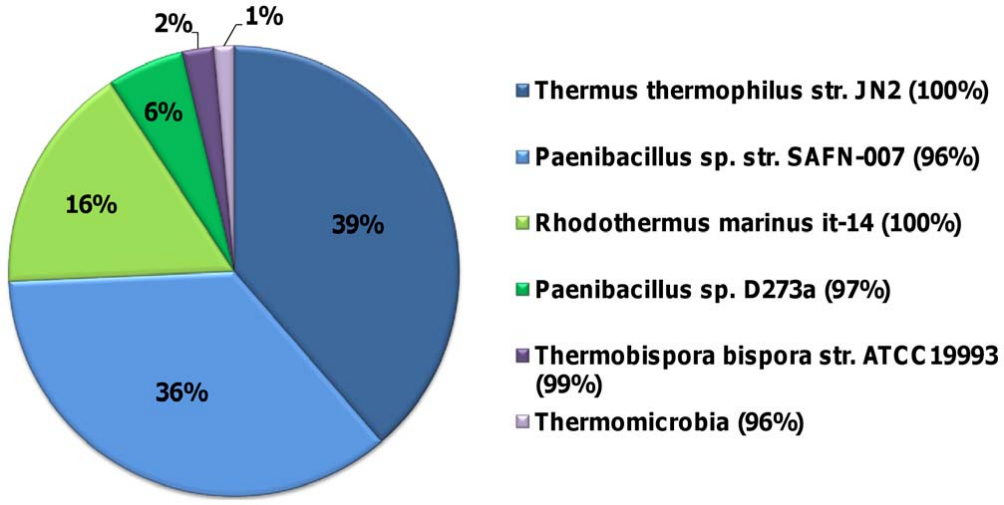
A pie chart showing the percent relative abundance of each taxon in the McCel-adapted thermophilic bacterial consortia. SSU pyrosequencing was conducted to identify community members. Only members with a relative abundance greater than 1% are reported. Relative abundance is calculated as a percentage of the total number of SSU reads for the community. The closest taxon to each organism in the community is reported in the legend. The percent identity between the consortial and closest taxon SSU sequence is in parentheses.

Previously, several thermophilic (60°C) switchgrass-adapted microbial communities were found to produce thermophilic glycoside hydrolases that were IL-tolerant [18]. One of these consortia was then perturbed with a variety of biomass-derived carbon sources and found to produce higher levels of endoglucanases when grown on microcrystalline cellulose (McCel) [24]. In this study, the enzymes produced by the McCel-adapted consortium were used as the endoglucanase component of an IL-tolerant enzymatic cocktail. The microbial community profile of this consortium was generated by amplicon pyrosequencing of SSU (small subunit) rRNA genes, and bacterial populations related to *Thermus thermophilus*, gram-positive thermophilic *Paenibacilli*, and *Rhodothermus marinus*, some of which are know biomass degraders, were found to compose 97% of the community (Figure 2) [25], [26], [27], [28].

A variety of glycoside hydrolase (GH) activities were detected in the supernatant recovered from the McCel-adapted consortium, including high levels of endoglucanase and xylanase activity (Table 1). MS-based proteomic measurements of the supernatant identified 124 proteins produced by the McCel-adapted consortium, with the most abundant proteins functionally grouping around sugar transport (ABC transporters), and the cell wall of gram positive bacteria (S-layer homology domain proteins). Proteins involved in sugar metabolism (glycoside hydrolases, sugar isomerases, sugar binding proteins, and sugar transporters) and oxidative stress (superoxide dismutase and catalase) were also frequent among the proteins detected in the supernatant (data not shown). The proteomics identified six glycoside hydrolase proteins in the supernatant, including 3 endoglucanases (GH5/9) and one cellobiohydrolase (GH48) (Table 2). A xylanase and an arabinofuranosidase were also identified. Mapping of these proteins to phylogenetic bins in metagenome demonstrated that they were expressed by the *Paenibacillus* population (see methods). In an attempt to correlate the activities measured in the supernatant with the proteomics, zymography was use to determine the number and molecular weight of endoglucanases and xylanases produced by the consortia (Figure S1). The supernatant harbored five or six major CMC-reactive bands and four xylan-reactive bands. Several of the glycoside hydrolases identified by the proteomics have predicted molecular weights that correspond to three of the major endoglucanases and one minor endoxylanases identified in the zymograms.

**Table 1:** Glycoside hydrolase activities produced by the thermophilic community.

Enzyme	Activity (U/ml)	STDEV
Endoglucanase	0.466	0.015
Cellobiohydrolase	0.014	0.001
β-D-glucosidase	0.041	0.001
Endoxylanase	1.319	0.013
α-L-arabinofuranosidase	0.210	0.010
β-D-xylosidase	0.018	0.001

Endoglucanase and endoxylanase activities were determined using the DNS assay on carboxymethyl cellulose or birtchwood xylan. Other activities were assessed using *p*-nitrophenyl substrates. U = µmol/min and is reported as the mean and standard deviation of triplicate experiments.

**Table 2:** Cellulase and xylanase from the thermophilic community identified by proteomics.

Predicted Function	Source Organism	IMG gene_oid	Protein (AA)	pfam	pfam info
Endoglucanase	*Paenibacillus*	2061998357	768	00759; 00942	Glyco_hydro_9; CBM_3
Endoglucanase	*Paenibacillus*	2062016312	542	00759; 02927	Glyco_hydro_9; CelD_N
1,4-beta-cellobiosidase	*Paenibacillus*	2062032019	770	02011	Glyco_hydro_48
Cellulase/beta-1,4-mannanase	No match	2061990256	518	00150; 00942	Glyco_hydro_5; CBM_3
Beta-1,4-xylanase	*Paenibacillus*	2061982776	581	00331; 02018	Glyco_hydro_10; CBM_4_9
Alpha-L-arabinofuranosidase	*Paenibacillus*	2061991733	496	6964	Alpha-L-AF_C

Predicted function of the proteins identified by proteomics is based on comparisons of the genes in the metagenome to the pfam, Clusters of Orthologous Groups (COGs), and the Kyoto Encyclopedia of Genes and Genomes (KEGG) databases. The IMG gene oid is the gene identifier for The Joint Genome Institute's Integrated Microbial Genomes database http://img.jgi.doe.gov/. The pfam assignment of the metagenome gene is indicated.

### Formulation of the JTherm cellulase cocktail

**Figure 3:**
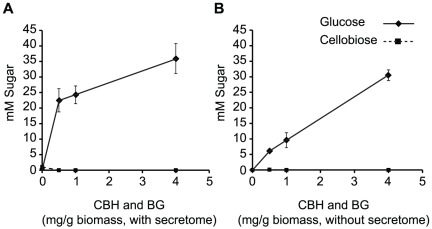
Enzymatic saccharification of IL-pretreated switchgrass by JTherm at 70°C pH 5.5. The supernatant from the thermophilic community at fixed concentration of 0.6× was augmented with various amounts of CBH and BG, and liberated glucose (♦) and cellobiose (▪) from IL-pretreated switchgrass were measured after 72 h incubation. Enzyme combinations were as follows: (A) supernatant, CBH, and BG; (B) CBH and BG without supernatant. The reaction was in a 1 ml volume with 25 mg of IL-pretreated switchgrass.

A cellulase cocktail requires a minimum of three types of glycoside hydrolase enzymes to efficiently liberate glucose from biomass: endoglucanase, cellobiohydrolase (CBH), and β-glucosidase (BG) [29]. The McCel-adapted consortium produced relatively low levels of CBH and BG activities (Table 1). To create a more efficient cellulase cocktail, the endoglucanase-rich supernatant recovered from the consortium was supplemented with a recombinant CBH (CBM3-GH5 of CelB) from *Caldicellulosiruptor saccharolyticus* and BG from *Thermotoga petrophila*. The activity of this cocktail, called JTherm, was validated by it's a ability to saccharify IL-pretreated switchgrass (Table 1; Figure 3) [30].

To find the optimal ratio of cellulase enzymes for JTherm to deconstruct IL-pretreated switchgrass, a fixed supernatant concentration from the McCel-adapted consortium (0.6×) was mixed with variable amounts of CBH and BG (0.5 to 4 mg of each enzyme/g biomass). These mixtures were tested for their ability to release glucose from the pretreated switchgrass at 70°C (Figure 3). The saccharification reactions were performed in M9TE medium (M9 minimal medium with added trace elements), which is commonly used for growth of *E. coli* to enable downstream microbial fuel production. While the supernatant alone released 0.4 mM glucose and 1 mM cellobiose from IL-pretreated switchgrass (4% of the total glucan), it released 22 mM of glucose (36% of the total glucan) when supplemented with 0.5 mg of CBH and BG/g of switchgrass (Figure 3A). In the absence of the supernatant, the same amount of CBH and BG released 4 times less glucose (6.1 mM), indicating a synergistic interaction between the glycoside hydrolase enzymes in the supernatant and the added recombinant enzymes (Figure 3B). At the highest CBH/BG loading tested (4 mg/g biomass) this synergistic relationship was lost; the levels of glucose release were similar to that of the two recombinant enzymes alone (36 mM vs. 30 mM glucose, respectively). However, a greater proportion of the total glucan content was converted to glucose (∼50 to 60%) at those enzyme loadings. Combining the supernatant with the CBH or BG individually released limited amounts (<10 mM) of glucose and cellobiose, indicating that both enzymes are required for efficient hydrolysis (data not shown).

### Tolerance of JTherm and CTec2 to temperature and ionic liquids

**Figure 4:**
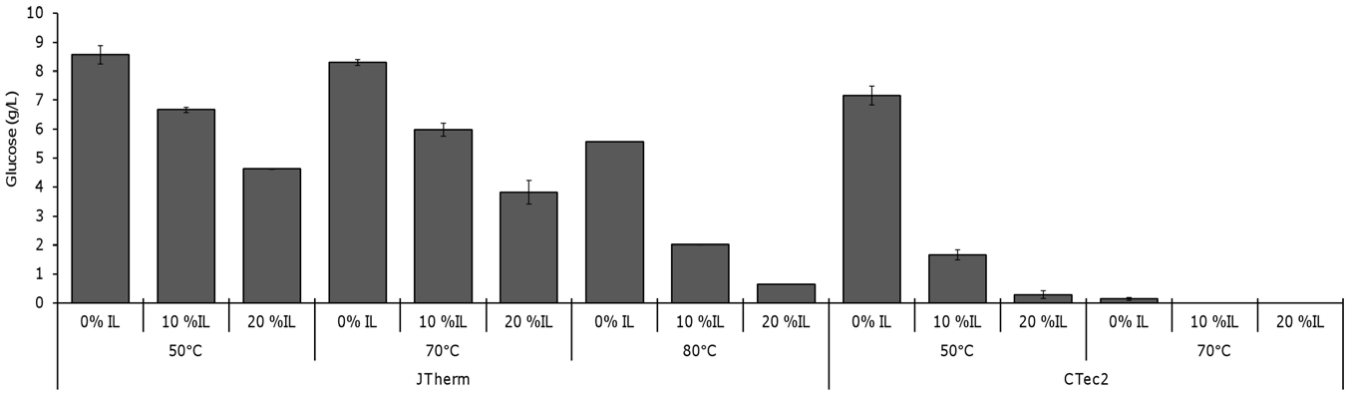
Activity of the JTherm and CTec2 cellulase cocktails on ionic-liquid pretreated switchgrass at various temperatures and in the presence of the ionic liquid [C2mim][OAc]. Samples were run at 2.5% w/v biomass loadings in 1 ml and incubated at pH 5.5 for 72 h with shaking.

The JTherm cocktail (formulated with 0.5 mg of BG and CBH/g biomass) and the commercial cocktail CTec2 (Novozymes) were profiled for their ability to liberate glucose from IL-pretreated switchgrass at high temperatures and in the presence of [C2mim][OAc] (Figure 4). The activity of the JTherm cocktail was highest at 50°C, yet it retained 97 and 65% of its activity at 70 and 80°C, respectively (Figure 4). JTherm was most active in the presence of ILs at 50°C (78 and 54% activity in 10 and 20% IL (w/v), respectively). This IL tolerance was slightly lower at 70°C, and JTherm even retained some activity in ILs at 80°C. The CTec2 cocktail however showed minimal activity at higher temperatures (70–80°C) or in the presence of ILs (23% activity in 10% (w/v) IL at 50°C).

### JTherm and CTec2 switchgrass hydrolysate analysis and FAEE biodiesel production

**Figure 5:**
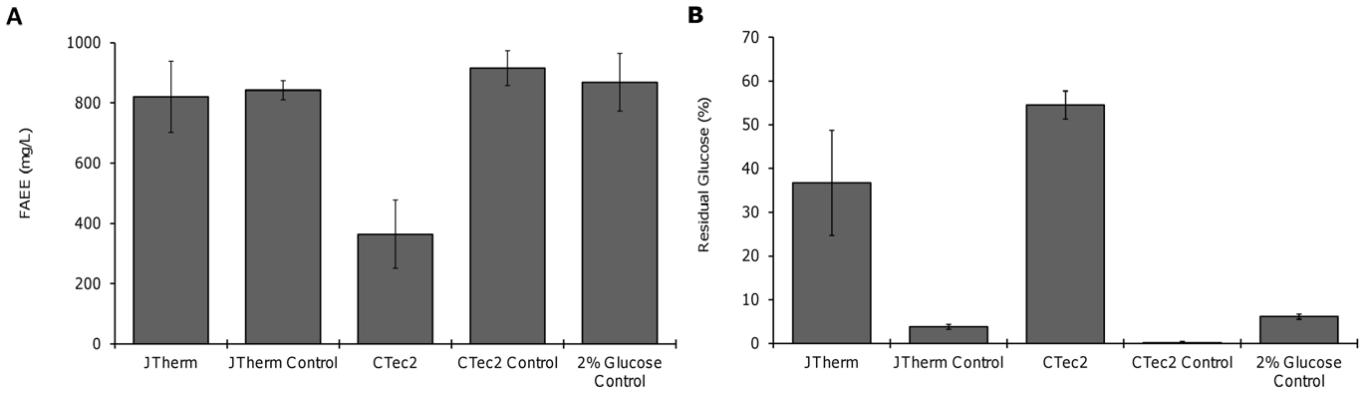
Biodiesel produced by an engineered *E. coli* strain fed hydrolysates derived from JTherm or CTec2 hydrolysis of IL-pretreated switchgrass. (A), and percentage of glucose remaining after fermentation (B). Glucose levels were adjusted to 2% for all hydrolysates and controls. The JTherm and CTec2 controls contained purified glucose and xylose at the same levels as their corresponding hydrolysate. No xylose was consumed during the fermentation (data not shown). Error bars indicate the standard deviation of triplicate experiments.

JTherm and CTec2 IL-pretreated switchgrass hydrolysates were tested for their ability to be converted into biofuel by a strain of *E. coli* engineered to produce FAEEs. To estimate the conversion efficiency of the hydrolysates, fuel production was compared to control medium containing only purified sugars, glucose and/or xylose (Figure 5) [5]. The hydrolysates were scaled up to produce sufficient amounts of sugars for microbial fuel production using higher biomass (10% w/v) and enzyme (1 mg of BG and CBH/g biomass for JTherm) loadings. Hydrolysates produced by JTherm at 70°C were light brown and those from CTec2 at 50°C were slightly darker brown. The fact that IL-pretreated switchgrass is brown in color suggests that, in addition to the sugars, other biomass-derived compounds are being released into the hydrolysates during saccharification. An absorbance scan of the hydrolysates was conducted to quantify this observation, and the CTec2 hydrolysate showed a greater overall absorbance between 280 and 500 nm, with peaks at 280 and 315 nm (Figure S2). The absorbance pattern of the hydrolysates showed some similarity to an organosolv purified lignin (Figure S2). The saccharifications were performed at 10% (w/v) biomass loadings, which were initially semi-solid but liquefied over the course of the 72 h incubation. CTec2 liquefied the biomass faster than JTherm (24 h vs. 48 h), and at the end of the reaction, the CTec2 treated biomass formed a more compact pellet than that produced by JTherm (28 vs 38% of the total saccharification volume). Sugar yields from the biomass indicated that CTec2 liberated 71% of the glucose and 100% of the xylose from the biomass, while JTherm liberated 48% of the glucose and 25% of the xylose (see methods). These observations indicated that of the two cocktails, CTec2 more thoroughly hydrolyzed the polysaccharides and deconstructed the biomass.

Comparison of the hydrolysates and control medium, each diluted to a final concentration of 2% glucose, revealed that the engineered *E. coli* strain could produce FAEE biodiesel from both hydrolysates, but not with equal efficiency. The JTherm hydrolysate produced equal amounts of FAEE as its control while the CTec2 hydrolysate produced 58% less FAEE than its control (Figure 5A). The *E. coli* strain did not completely utilize the glucose in either hydrolysate, while the all sugar was consumed in the control medium (Figure 5B). After fermentation, 55% of the glucose remained in the CTec2 hydrolysate and 37% of the glucose remained in the JTherm hydrolysate. These results indicate that both the hydrolysates may contain inhibitors, yet they appear to be present at lower levels in the JTherm hydrolyzate where fuel production is not impaired.

### Inhibition of FAEE production by [C2mim][OAc]

**Figure 6:**
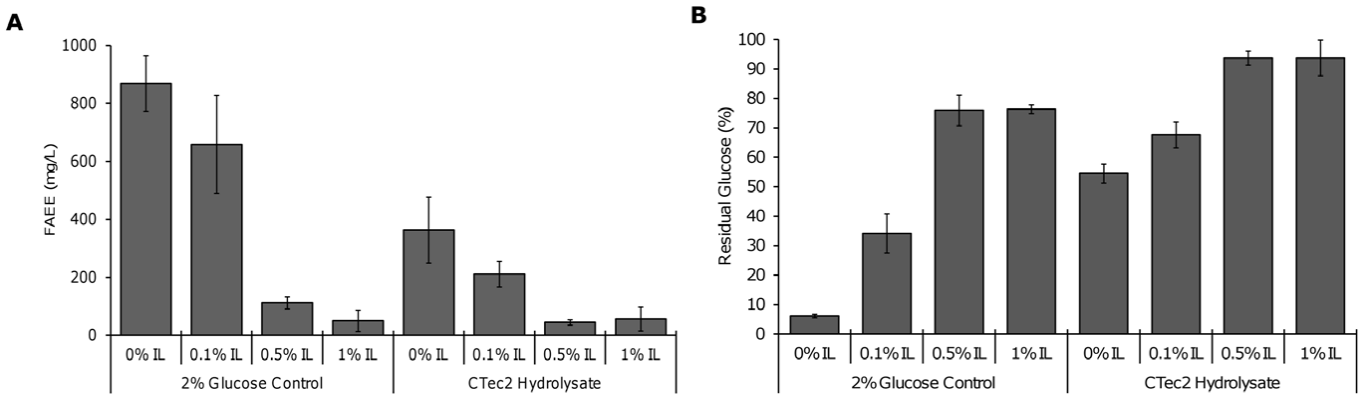
Effects of ionic liquids on biodiesel production by an engineered *E. coli* strain. The strain was fed either 2% glucose or a CTec2 hydrolysate of IL- pretreated switchgrass containing 0–1% (w/v) [C2mim][OAc] [(A). The percentage of glucose remaining after fermentation was measured (B). Glucose levels were adjusted to 2% for all hydrolysates and controls. The CTec2 control contained equivalent amounts of purified glucose and xylose as the hydrolysate. No xylose was consumed during the fermentation (data not shown). Error bars indicate the standard deviation of triplicate experiments.

Residual [C2mim][OAc] remaining in the biomass after pretreatment has been shown to inhibit growth in *S. cerevisiae* at concentrations as low as 0.3% (w/v) in the hydrolysate [31]. The JTherm and CTec2 hydrolysates contained only 0.05% and 0.07% (w/v) [C2mim][OAc], respectively, so it is unclear whether such low IL concentrations would inhibit *E. coli* growth and fuel production (see methods). An analysis of the inhibitory effect of [C2mim][OAc] on FAEE production in *E. coli* revealed that IL concentrations as low as 0.1% (w/v) partially inhibit FAEE production (25% reduction) and sugar consumption (Figure 6A and B). However, this inhibitory effect was too mild to explain the concomitant reduction in FAEE production (58%) and sugar consumption seen in the CTec2 hydrolysate, indicating the presence of additional inhibitory compounds in the hydrolysate.

### Inhibition of *E. coli* growth by the CTec2 hydrolysate

**Figure 7:**
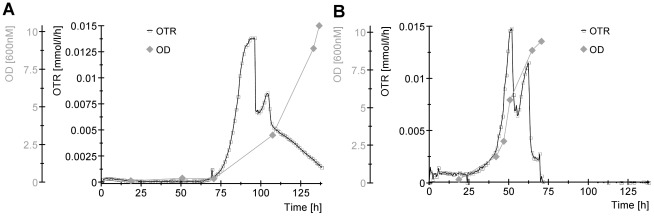
Growth of an *E. coli* strain engineered to produce biodiesel on CTec2 hydrolysate (A) and control sample containing 2% glucose and 1% xylose (B). Oxygen transfer rate (OTR), and cell density (OD_600_) were monitored during the fermentation to determine the impacts of the hydrolysate on growth and respiration.

To gain a better understanding of the mechanisms behind the inhibition of FAEE production, growth, sugar consumption and respiration of the FAEE producing *E. coli* strain were monitored during fermentation of the CTec2 hydrolysate, as it exhibited the strongest inhibitory effect (Figure S4). Comparison of cell densities and oxygen transfer rates (OTR) between the CTec2 hydrolysate and controls shows that the *E. coli* strain has a pronounced lag phase (40 h longer) in the hydrolysate (Figure 7A and B). Once growth begins, the maximum oxygen utilization rate is slightly higher in the hydrolysate versus the control (0.174 vs 0.118 h^−1^; based on the first respiration peak). However, growth in the CTec2 culture appears to lag behind the oxygen utilization rate, with cell density reaching an OD_600_ of only 3.0 after the second respiration peak in the CTec2 culture versus 9.5 in the control, indicating a slower growth rate. Eventually the cells in the CTec2 hydrolysate reach a maximum density similar to the control, but it takes much longer to do so (70 vs 50 h after the beginning of exponential growth).

## Discussion

In this study, we developed a thermo- and IL-tolerant cellulase cocktail, JTherm, that liberates sugars from biomass in the presence of up to 20% ionic liquid. A correlation between thermotolerance and IL tolerance has been established for purified thermophilic glycoside hydrolases, and this correlation was used to develop an ionic liquid-tolerant cellulase cocktail [18], [20]. This correlation was confirmed for native thermophilic endoglucanase and xylanase enzymes produced by the bacterial consortia in this study, as well as the recombinant BG and CBH, derived from two different thermophilic bacteria. The combination of these enzymes produced a cellulase cocktail able to withstand IL concentrations that completely inactivated a commercial fungal-derived cellulase cocktail.

Although it is not clear why the correlation between thermotolerance and IL-tolerance exists, these data suggest that IL-tolerance may be a general characteristic of thermophilic enzymes and that the use of these enzymes is one potential route to generating commercial IL-tolerant cellulase cocktails. The JTherm cocktail provides an exemplary demonstration, but higher titers of these enzymes will be required to scale up saccharification with this cocktail. Commercialization would also require the identification of the most important enzymes produced by the bacterial community, and optimization of a recombinant expression system to produce high titers of these enzymes. It will be useful to compare a purely recombinant bacterial cellulase cocktail to JTherm to determine whether a limited subset of enzymes is as effective at deconstructing biomass as a native cocktail, as has been done for the components of the cellulase cocktail in *Trichoderma reesei* [32]. Comparison of the native mixture of glycoside hydrolases with a purely recombinant cocktail will also indicate whether bacterial accessory enzymes are present that may account for the synergistic activities observed at low CBH and BG loadings.

The JTherm cocktail functions relatively well in 20% (w/v) [C2mim]OAc, between 50 and 70°C, but its activity was reduced by approximately 50% at 70°C, indicating that it is unlikely to function well at much higher IL concentrations. This observation limits the levels of ILs that can be used for pretreatment, or requires the addition of water to the IL-pretreated biomass prior to saccharification with JTherm. However, studies have shown that aqueous IL solutions close to 20% IL can be used to pretreat biomass, indicating that this limit is not a major barrier to combining enzymatic saccharification with pretreatment, as outlined in the configuration in Figure 1B [15]. Even if pure ILs are used for pretreatment, having enzymes that are active in a 20% IL solution may reduce the amount of water needed to wash the biomass after pretreatment with ILs. In this study, significant amounts of water and ethanol were used to remove ILs from the biomass prior to saccharification, yet there was still about 0.5% IL left in the biomass. Any fewer washes would likely lead to issues with enzyme inhibition during saccharification (for commercial fungal enzymes) or downstream inhibition of microbial fuel production, as we showed that even 0.1% w/v IL begins to inhibit fuel production in *E. coli*. The configuration in Figure 1B would circumvent washing altogether by extracting sugars liberated from biomass by JTherm with chemical or physical separation techniques, which may also remove any biomass-derived inhibitors. Now that the JTherm cocktail has been formulated and validated, this alternate configuration may be tested in future studies, where the economic viability of each bioprocessing configuration can be assessed and compared.

A successful biomass-to-biofuel bioprocessing configuration requires the efficient microbial conversion of hydrolysates of pretreated biomass into fuel. Biomass-deconstructing enzyme cocktails are commonly used to saccharify pretreated biomass but they have the potential to liberate inhibitor compounds from the biomass along with the sugars, and these compounds can have a deleterious effect on biofuel production [33]. The JTherm and CTec2 cocktails liberate sugars from IL-pretreated switchgrass, but the hydrolysates appear to be contaminated with low levels of ILs and possibly other biomass-derived inhibitors that together can inhibit growth and reduce fuel production [14]. The fact that JTherm did not exhibit lower FAEE production indicates that this enzyme cocktail does not release significant amounts of these inhibitors into the hydrolysate. In contrast, the pronounced lag phase that occurs in the CTec2 hydrolysate indicates transient inhibition of the *E. coli* strain, which is consistent with the presence of biomass-derived inhibitors that may force the cells to undergo a stringent response and adapt to the inhibitors before growth can occur [33], [34], [35]. The lower FAEE production in the CTec2 hydrolysate compared to JTherm and control medium may therefore be a consequence of diversion of cellular energy and resources towards detoxification, i.e. through the use of active efflux pumps to remove inhibitors from the cell, and away from cell growth and fuel production [36], [37]. Further detailed characterization of the cellular response to these hydrolysates will be needed to understand the mechanisms behind the transient inhibition and reduced fuel production.

In summary, we generated an IL-tolerant cellulase cocktail to enable the development of efficient and potentially inexpensive IL-pretreatment bioprocessing configurations that may reduce barriers to commercialization (i.e. simultaneous IL-pretreatment and saccharification), and examined the feasibility of using IL-pretreated lignocellulosic biomass to produce biofuels. A thermophilic and IL-tolerant cellulase cocktail, called JTherm, was constructed by combining native and recombinant enzymes derived from thermophilic bacteria. This cellulase cocktail stands alone in its ability to function so efficiently in the presence of [C2mim][OAc], one of the most potent ionic liquids used for biomass pretreatment. Several other studies have investigated IL-tolerant cellulase cocktails, but those studies either use ILs that pretreat biomass less effectively than [C2mim][OAc] or excessive amounts of celllulase enzymes, both of which are unlikely to lead to commercially viable technologies [8], [38], [39]. In addition, to explore the possibility of whether microbial inhibitors could be generated during enzymatic hydrolysis of IL-pretreated biomass, hydrolysates were generated by JTherm and CTec2 and fed to an *E. coli* strain engineered to produce FAEE biodiesel. To our knowledge, this is the first report of the production of an advanced biofuel from IL-pretreated biomass using a metabolically engineered organism. Like *S. cerevisiae*, *E.coli* is inhibited by ILs, and by unidentified, possibly biomass-derived, inhibitors [31]. This study both confirms the feasibility of using IL-pretreatment to produce biofuels and shows that thermotolerant enzymes can be used to develop IL-tolerant enzymes cocktails that can potentially lead to the development of inexpensive IL-based biomass-to-biofuels technologies.

## Materials and Methods

### Cultivation of the thermophilic community and supernatant preparation

The thermophilic bacterial community was grown on microcrystalline cellulose in M9TE minimal medium as described previously [18], [24]. Briefly, a thermophilic bacterial community cultivated on switchgrass at 60°C was used to inoculate a 50 ml culture containing 1% (w/v) microcrystalline cellulose in M9 minimal medium supplemented with trace elements. The culture was then incubated for 2 weeks at 60°C with shaking at 200 rpm. This community was maintained by serial passage (1∶25 dilution) under the same conditions. The complement of proteins in the supernatant was prepared by removing all insoluble material (biomass and microcrystalline cellulose, which were saved for DNA isolation) from the culture by centrifugation at 21,000× *g* for 5 minutes, followed by filtration of the supernatant through a 0.2 µm filter. The supernatant was then aliquoted into 1 mL volumes, lyophilized, and stored at −80°C.

### DNA isolation and SSU rRNA pyrosequencing

The solid fraction from 8 mL of the McCel culture containing the thermophilic bacterial community was collected in 2 mL lysing matrix E tubes (MP Bio # 116914050) and frozen at −80°C. DNA isolation and SSU pyrosequencing were performed as previously described [18].

### Proteomics

Aliquots of the thermophilic community supernatant proteins were split into two fractions and either pelleted or separated by size on denaturing SDS PAGE gels. The Coomassie-stained bands from the gels were excised and digested with trypsin by using established procedures [40] and the protein pellet was digested with trypsin using the following procedure [41]. Peptide sequences were identified by liquid chromatography-mass spectrometry methods described previously [42]. Peptides were identified by using the MASCOT search algorithm searched against sequences from a metagenome of the parent switchgrass-adapted culture (metagenome manuscript in preparation)^16^. Briefly, metagenome shotgun sequence data was sequenced, assembled and annotated at the DOE Joint Genome Institute (sequences are available at the JGI IMG/M Genomes site using the Taxon Object ID 2061766001). Contiguous sequences containing phylogenetic markers were used to identify phylogenetic bins, and the remaining contigs were mapped to these bins using ClaMS (http://clams.jgi-psf.org/). The bins were used to predict the source organism of the peptides identified by mass spectrometry. Proteins were considered to have a positive identification if they had at least two matching peptides.

### Zymography

Zymograms were generated as previously described [18]. A volume of 10 µL of the supernatant was run in each zymogram and the enzyme reaction was conducted in 50 mM sodium acetate buffer pH 5.0 at 70°C for 30 to 120 minutes. Zymograms were stained with 0.1% Congo red dye and negative images were taken to highlight the clearing zones.

### Ionic liquid pretreatment of switchgrass

Switchgrass was pretreated with the ionic liquid 1-ethyl-3-methylimidazolium acetate ([C2mim][OAc]) at a 10∶1 w/w ratio at 120°C for 3 h. The biomass was recovered by adding an equal volume of deionized water, and then washed three times with an equal volume of water, once with an equal volume of ethanol, and once more with an equal volume of water. The biomass was then dried by lyophilization and used directly in saccharification experiments. The IL-pretreated switchgrass was composed of 41% glucan and 13% xylan, determined as previously described [43]. The amount of [C2mim][OAc] remaining in the switchgrass was estimated using the measured IL concentrations in the hydrolyzate after saccharification (see methods below); the IL concentration in the hydrolyzate was determined to be 0.05%, and at 10% w/v loadings of IL-pretreated biomass in the saccharification that would equal approximately 0.5% IL left in the biomass after pretreatment.

### JTherm cellulase cocktail formulation

The lyophilized supernatant was reconstituted in M9TE at pH 5.5. The recombinant cellobiohydrolase (CBH) was a truncated construct of CelB (CBM3-GH5) from *C. saccharolyticus* and the β-glucosidase (BG) from *T. petrophia* (UniProt ID: A5IL97) were expressed in *E. coli* with a C-terminal His(6×)-tag in the pDEST42 expression vector (Invitrogen; Carlsbad, CA), and purified as described previously by using IMAC and anion exchange chromatography [30]. The purified recombinant enzymes were buffer exchanged in M9TE at pH 5.5 using a DG10 desalting column (Bio-Rad, Hercules, CA). The performance of enzymatic saccharification of IL-pretreated switchgrass by JTherm was tested by mixing various amounts of CBH and BG (12.5 µg to 100 µg of each) to 0.6× concentration of the supernatant in 1 ml reaction volume (0.6 mL of reconstituted supernatant) containing 25 mg of IL-pretreated switchgrass. The enzymatic saccharification was done at 70°C for 72 hr with constant shaking. The pH of the M9TE reaction buffer was set at 5.5, because it drops to near 5.0 during the saccharification. The hydrolyzate was separated by centrifugation at 14,000× g for 10 min followed by syringe filtration. The amount of cellobiose and glucose released in the hydrolyzate was measured by Agilent 1100 series HPLC equipped with an Aminex HPX-87H ion exchange column (Biorad), using 4 mM H_2_SO_4_ as solvent, a flow rate of 0.6 ml min^−1^ and a column temperature of 50°C. Sugars were detected with Agilent 1200 series DAD and RID detectors.

### JTherm cellulase cocktail thermostability and tolerance to ionic liquids

Enzymatic saccharification of IL-pretreated switchgrass by the JTherm cocktail (0.6× supernatant, and 0.5 mg/g biomass of each purified recombinant CBH and BG) was tested at 50, 70, and 80°C, and in 10 or 20% (w/v) of the ionic liquid [C2mim]OAc. The CTec2 cellulase cocktail kindly provided by Novozymes (Davis, CA) was used as a control and was loaded at 12 mg enzyme product/g biomass. A dose curve for CTec2 was generated to determine optimal enzyme loading using the same setup described below at 50°C (Figure S3). Each reaction contained 1 mL of M9TE at pH 5.5 (the pH was adjusted with HCl) with 0, 10, or 20% (w/v) ionic liquid, enzyme, and 25 mg IL-pretreated switchgrass. The pH of the medium was initially set at 5.5 due to the observation that the pH drops during saccharification to around 5.0, which is the optimal pH for CTec2. The JTherm cocktail functions well between pH 5.0 and 7.0, so its initial pH was also set to 5.5 to simplify the experimental design. The enzymes and the other components of the reaction were preheated separately at 50, 70, or 80°C for 10 minutes before mixing and the reaction was shaken for 72 hr at the appropriate temperature. Levels of sugars produced in the hydrolysate were measured using HPLC as described above.

### Saccharification of IL-pretreated switchgrass

IL-pretreated switchgrass hydrolysates were generated to determine whether they would support growth of an *E. coli* strain engineered to produce fatty acid ethyl-ester (FAEE) biodiesel [5]. The hydrolysates were generated in a 10 mL scale with 1 g of IL-pretreated switchgrass in M9TE medium pH 5.5. Since the biomass loadings were increased to 10% w/v, the BG and CHB enzyme loadings were increased to 1 mg/g biomass for JTherm and 15 mg EP/g biomass for CTec2. The JTherm reaction was performed at 70°C and the CTec2 at 50°C. The JTherm generated a hydrolysate with 2.1% glucose and 0.36% xylose, while the CTec2 cocktail generated a hydrolysate with 3% glucose and 1.6% xylose. The hydrolysates were then centrifuged at 6,000 g for 10 minutes (the volume the biomass pellet occupied was measured to determine the extent of biomass degradation), the supernatant was passed through a 0.2 µm filter, and the pH was adjusted to 7.0 with KOH (generating KCl salt). Next, 50 µL of 1.5 M MOPS pH 7.4 and 30 µL of 50 mg/mL NH_4_Cl were added per 1 mL of supernatant, and the entire solution was filter sterilized. The CTec2 hydrolysate was diluted to 2% glucose 1% xylose in the same medium. Control M9TE medium was made containing all chemicals above, 2% glucose, and either zero, 0.4, or 1% xylose. Sugar levels before and after fermentation were measure by HPLC as described above.

### FAEE biofuel production from hydrolysates using an *E. coli* biocatalyst

The FAEE producing *E. coli* strain [5] was adapted to the 2% glucose control M9TE containing 50 µg/mL chloramphenicol, 100 µg/mL carbenicillin, 5 µg/mL tetracycline, and 1 µg/mL thiamine overnight at 37°C with shaking at 200 rpm. Cells were grown to an OD_600_ of between 0.5 and 0.8, pelleted by centrifugation at 6,000 g at 25°C, and resuspended to an OD_600_ of 1.0 in the aforementioned hydrolyzates and control medium, with additional 25 µM IPTG, 150 µg/mL chloramphenicol, 200 µg/mL carbenicillin, 15 µg/mL tetracycline, and 0.1 ng/mL thiamine. To test the effects of IL on fermentation, [C2mim]OAc was added to CTec2 hydrolyzate or 2% glucose control medium samples at 0.1, 0.5, and 1% (w/v). Cultures were grown in 2 mL aliquots in triplicate at 37°C with shaking.

For growth curves and OTR measurements, CTec2 hydrolysates were prepared in duplicates of 35 mL volume in a similar manner with the following exceptions: Korz medium lacking citrate was used instead of M9TE, 2 M MOPS pH 6.9 was added to a final concentration of 200 mM, and NH_4_SO_4_ was substituted for NH_4_Cl to a final concentration of 8 g/L. Control media contained 2% glucose 1% xylose with or without added CTec2 enzyme product at the same loadings used for saccharification, and 2% glucose. The hydrolysates were inoculated with *E. coli* as described above, except at an OD_600_ of 0.05, and grown in a Respiration Activity Monitoring System (RAMOS) [44] system within a Kuhner labterm LT-X shaker. Cultures were grown at 37°C and shaken at 250 rpm with a shaking diameter of 50 mm. Maximum measurement time was set at 25 min and rinsing time set at 10 min with 10 ml/min air flow. Samples for sugar and OD_600_ measurements were taken at several time points. Maximum respiration rate was calculated as ln(OTR_b_/OTR_a_)/(t_b_−t_a_), where t_a_ and t_b_ are the times at the beginning and end of exponential growth, respectively and OTR_a_ and OTR_b_ are the oxygen transfer rates at t_a_ and t_b_, respectively.

### [C2mim][OAc] concentration measurements in the hydrolysates

The concentration of [C2mim][OAc] in the hydrolysates was determined using an HPLC and LC/MSD Quad SL system (Agilent Technologies Inc., Santa Clara, CA) as described previously [31]. The concentration in % (w/v) units was 0.07±0.02 in the CTec2 hydrolysate, 0.05±0.01 in the JTherm hydrolysate, and 0.07±0.03 in the CTec2-Korz medium hydrolysate used for growth curves (see above).

## Supporting Information

Figure S1 **Zymography of the Endoglucanase (A) and endoxylanase (B) enzymes produced by the thermophilic community.** Gels were embedded with carboxymethyl cellulose or soluble birchwood xylan and enzyme reactions were run at pH 5.0 and 70°C for 30 minutes to 2 h. Substrate clearing zones created by enzymatic digestion are black. Molecular weight markers are in kilodaltons.
(DOC)

Figure S2 **UV/Vis absorbance scans of the Hydrolysates.** JTherm (from Figure 6) and CTec2 (from Figure 8) IL-pretreated switchgrass hydrolysates were diluted 20 fold and the water soluble fraction of organosolv lignin (Sigma #371033) was at 5 mg/ml. The hydrolysates and lignin have an absorbance peak at around 280 nm, and the hydrolysates have a second absorbance peak at around 315 nm. The CTec2 hydrolysate has a greater overall absorbance than JTherm, indicating higher concentrations of biomass-derived compounds are present in the CTec2 hydrolysate.
(DOC)

Figure S3 **Dose curve of the CTec2 cellulase cocktail on ionic-liquid pretreated switchgrass in M9 salts pH 5.0 at 50°C.** The CTec2 dose is reported mg of enzyme product added per gram of total solids based on triplicate samples.
(DOC)

Figure S4 **Growth of an** ***E. coli*** **strain engineered to produce biodiesel on CTec2 hydrolysate (A) and control samples containing 2% glucose and 1% xylose (B), 2% glucose and 1% xylose with the CTec2 enzyme product (C), and 2% glucose (D).** Oxygen transfer rate (OTR), cell density (OD_600_), and sugar concentration were monitored during the fermentation to determine the impacts of the hydrolysate on growth and respiration.
(DOC)

## References

[1] Pimentel D (2003). Ethanol Fuels: Energy Balance, Economics, and Environmental Impacts Are Negative.. Natural Resources Research.

[2] Laboratory ORN (2011). U.S. Billion-Ton Update: Biomass Supply for a Bioenergy and Bioproducts Industry. US DOE Energy Efficiency and Renewable Energy web site.. http://www1eereenergygov/biomass/pdfs/billion_ton_updatepdf.

[3] Decker J (2009). Going against the grain: Ethanol from lignocellulosics.. Renewable Energy World Magazine.

[4] Peralta-Yahya PP, Keasling JD (2010). Advanced biofuel production in microbes.. Biotechnol J.

[5] Steen EJ, Kang Y, Bokinsky G, Hu Z, Schirmer A (2010). Microbial production of fatty-acid-derived fuels and chemicals from plant biomass.. Nature.

[6] DOE US (2010). Using Fermentation and Catalysis to Make Fuels and Products: BIOCHEMICAL CONVERSION. US DOE Energy Efficiency and Renewable Energy web site.. http://www1eereenergygov/biomass/pdfs/biochemical_four_pagerpdf.

[7] Wald ML (2011). U.S. Backs Project to Produce Fuel From Corn Waste..

[8] Tadesse H, Luque R (2011). Advances on biomass pretreatment using ionic liquids: An overview..

[9] Zhao H, Jones CIL, Baker GA, Xia S, Olubajo O (2009). Regenerating cellulose from ionic liquids for an accelerated enzymatic hydrolysis.. Journal of Biotechnology.

[10] Li C, Knierim B, Manisseri C, Arora R, Scheller HV (2010). Comparison of dilute acid and ionic liquid pretreatment of switchgrass: Biomass recalcitrance, delignification and enzymatic saccharification.. Bioresource Technology.

[11] Klein-Marcuschamer D, Simmons BA, Blanch HW (2011). Techno-economic analysis of a lignocellulosic ethanol biorefinery with ionic liquid pre-treatment.. Biofuels, Bioproducts and Biorefining.

[12] Turner MB, Spear SK, Huddleston JG, Holbrey JD, Rogers RD (2003). Ionic liquid salt-induced inactivation and unfolding of cellulase from Trichoderma reesei.. Green Chemistry.

[13] Lee S-M, Chang W-J, Choi A-R, Koo Y-M (2005). Influence of ionic liquids on the growth of Escherichia coli.. Korean Journal of Chemical Engineering.

[14] Ganske F, Bornscheuer UT (2006). Growth of Escherichia coli, Pichia pastoris and Bacillus cereus in the presence of the ionic liquids [BMIM][BF4] and [BMIM][PF6] and Organic Solvents.. Biotechnol Lett.

[15] Fu D, Mazza G (2011). Aqueous ionic liquid pretreatment of straw.. Bioresource Technology.

[16] Brennan T, Datta S, Blanch H, Simmons B, Holmes B (2010). Recovery of Sugars from Ionic Liquid Biomass Liquor by Solvent Extraction.. BioEnergy Research.

[17] Ahring BK, Klinke HB, Thomsen AB (2004). Inhibition of ethanol-producing yeast and bacteria by degradation products produced during pre-treatment of biomass.. Applied Microbiology and Biotechnology.

[18] Gladden JM, Allgaier M, Miller CS, Hazen TC, VanderGheynst JS (2011). Glycoside hydrolase activities of thermophilic bacterial consortia adapted to switchgrass.. Appl Environ Microbiol.

[19] Zhang T, Datta S, Eichler J, Ivanova N, Axen SD (2011). Identification of a haloalkaliphilic and thermostable cellulase with improved ionic liquid tolerance.. Green Chemistry.

[20] Datta S, Holmes B, Park JI, Chen ZW, Dibble DC (2010). Ionic liquid tolerant hyperthermophilic cellulases for biomass pretreatment and hydrolysis.. Green Chemistry.

[21] Nakayama S, Kiyoshi K, Kadokura T, Nakazato A (2011). Butanol Production from Crystalline Cellulose by Cocultured Clostridium thermocellum and Clostridium saccharoperbutylacetonicum N1–4.. Applied and Environmental Microbiology.

[22] Kundu S, Ghose TK, Mukhopadhyay SN (1983). Bioconversion of Cellulose into Ethanol by Clostridium-Thermocellum – Product Inhibition.. Biotechnology and Bioengineering.

[23] Shaw AJ, Podkaminer KK, Desai SG, Bardsley JS, Rogers SR (2008). Metabolic engineering of a thermophilic bacterium to produce ethanol at high yield.. Proceedings of the National Academy of Sciences.

[24] Gladden JM, Eichorst SA, Hazen TC, Simmons BA, Singer SW (2012). Substrate perturbation alters the glycoside hydrolase activities and community composition of switchgrass-adapted bacterial consortia.. Biotechnol Bioeng.

[25] Watanabe K, Nagao N, Yamamoto S, Toda T, Kurosawa N (2007). Thermobacillus composti sp. nov., a moderately thermophilic bacterium isolated from a composting reactor.. Int J Syst Evol Microbiol.

[26] Touzel JP, O'Donohue M, Debeire P, Samain E, Breton C (2000). Thermobacillus xylanilyticus gen. nov., sp. nov., a new aerobic thermophilic xylan-degrading bacterium isolated from farm soil.. Int J Syst Evol Microbiol 50 Pt.

[27] Bjornsdottir SH, Blondal T, Hreggvidsson GO, Eggertsson G, Petursdottir S (2006). Rhodothermus marinus: physiology and molecular biology.. Extremophiles.

[28] Lyon PF, Beffa T, Blanc M, Auling G, Aragno M (2000). Isolation and characterization of highly thermophilic xylanolytic Thermus thermophilus strains from hot composts.. Canadian Journal of Microbiology.

[29] Meyer AS, Rosgaard L, Sørensen HR (2009). The minimal enzyme cocktail concept for biomass processing.. Journal of Cereal Science.

[30] Park JI, Kent MS, Datta S, Holmes BM, Huang Z (2011). Enzymatic hydrolysis of cellulose by the cellobiohydrolase domain of CelB from the hyperthermophilic bacterium Caldicellulosiruptor saccharolyticus.. Bioresource Technology.

[31] Ouellet M, Datta S, Dibble DC, Tamrakar PR, Benke PI (2011). Impact of ionic liquid pretreated plant biomass on Saccharomyces cerevisiae growth and biofuel production..

[32] Banerjee G, Car S, Scott-Craig JS, Borrusch MS, Aslam N (2010). Synthetic enzyme mixtures for biomass deconstruction: Production and optimization of a core set.. Biotechnology and Bioengineering.

[33] Klinke HB, Thomsen AB, Ahring BK (2004). Inhibition of ethanol-producing yeast and bacteria by degradation products produced during pre-treatment of biomass.. Applied Microbiology and Biotechnology.

[34] Zaldivar J, Martinez A, Ingram LO (1999). Effect of selected aldehydes on the growth and fermentation of ethanologenic Escherichia coli.. Biotechnology and Bioengineering.

[35] Chang DE, Smalley DJ, Conway T (2002). Gene expression profiling of Escherichia coli growth transitions: an expanded stringent response model.. Molecular Microbiology.

[36] Higgins CF (2001). ABC transporters: physiology, structure and mechanism – an overview.. Research in Microbiology.

[37] Nikaido H, Zgurskaya HI (2001). AcrAB and related multidrug efflux pumps of Escherichia coli.. Journal of Molecular Microbiology and Biotechnology.

[38] Li Q, Jiang X, He Y, Li L, Xian M (2010). Evaluation of the biocompatibile ionic liquid 1-methyl-3-methylimidazolium dimethylphosphite pretreatment of corn cob for improved saccharification.. Applied Microbiology and Biotechnology.

[39] Wang Y, Radosevich M, Hayes D, Labbé N (2011). Compatible Ionic liquid-cellulases system for hydrolysis of lignocellulosic biomass.. Biotechnology and Bioengineering.

[40] Sweetlove LJ, Heazlewood JL, Herald V, Holtzapffel R, Day DA (2002). The impact of oxidative stress on Arabidopsis mitochondria.. Plant Journal.

[41] Shevchenko A, Tomas H, Havlis J, Olsen JV, Mann M (2007). In-gel digestion for mass spectrometric characterization of proteins and proteomes.. Nat Protocols.

[42] Chhabra SR, Joachimiak MP, Petzold CJ, Zane GM, Price MN (2011). Towards a Rigorous Network of Protein-Protein Interactions of the Model Sulfate Reducer *Desulfovibrio vulgaris* Hildenborough.. PLoS One.

[43] Sluiter BH A, Ruiz R, Scarlata C, Sluiter J, Templeton D, Crocker D (2011). Determination of Structural Carbohydrates and Lignin in Biomass.. National Renewable Energy Laboratory Technical Report NREL/.

[44] Anderlei T, Zang W, Papaspyrou M, Büchs J (2004). Online respiration activity measurement (OTR, CTR, RQ) in shake flasks.. Biochemical Engineering Journal.

