# Supplementary material for: A Thermophilic Ionic Liquid-Tolerant Cellulase Cocktail for the Production of Cellulosic Biofuels
Source: PLoS One. 2012 May 23;7(5):e37010. doi: 10.1371/journal.pone.0037010 (PMC3359315; doi:10.1371/journal.pone.0037010)
Supplement: Figure S1 — Zymography of the Endoglucanase (A) and endoxylanase (B) enzymes produced by the thermophilic community. Gels were embedded with carboxymethyl cellulose or soluble birchwood xylan and enzyme reactions were run at pH 5.0 and 70°C for 30 minutes to 2 h. Substrate clearing zones created by enzymatic digestion are black. Molecular weight markers are in kilodaltons. (DOC) [file pone.0037010.s001.doc]

**
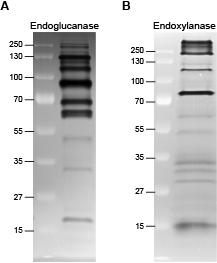
**

**Figure S1. Zymography of the Endoglucanase (A) and endoxylanase (B) enzymes produced by the thermophilic community.** Gels were embedded with carboxymethyl cellulose or soluble birchwood xylan and enzyme reactions were run at pH 5.0 and 70°C for 30 minutes to 2h. Substrate clearing zones created by enzymatic digestion are black. Molecular weight markers are in kilodaltons.
