# Supplementary material for: A Thermophilic Ionic Liquid-Tolerant Cellulase Cocktail for the Production of Cellulosic Biofuels
Source: PLoS One. 2012 May 23;7(5):e37010. doi: 10.1371/journal.pone.0037010 (PMC3359315; doi:10.1371/journal.pone.0037010)
Supplement: Figure S2 — UV/Vis absorbance scans of the Hydrolysates. JTherm (from Figure 6) and CTec2 (from Figure 8) IL-pretreated switchgrass hydrolysates were diluted 20 fold and the water soluble fraction of organosolv lignin (Sigma #371033) was at 5 mg/ml. The hydrolysates and lignin have an absorbance peak at around 280 nm, and the hydrolysates have a second absorbance peak at around 315 nm. The CTec2 hydrolysate has a greater overall absorbance than JTherm, indicating higher concentrations of biomass-derived compounds are present in the CTec2 hydrolysate. (DOC) [file pone.0037010.s002.doc]

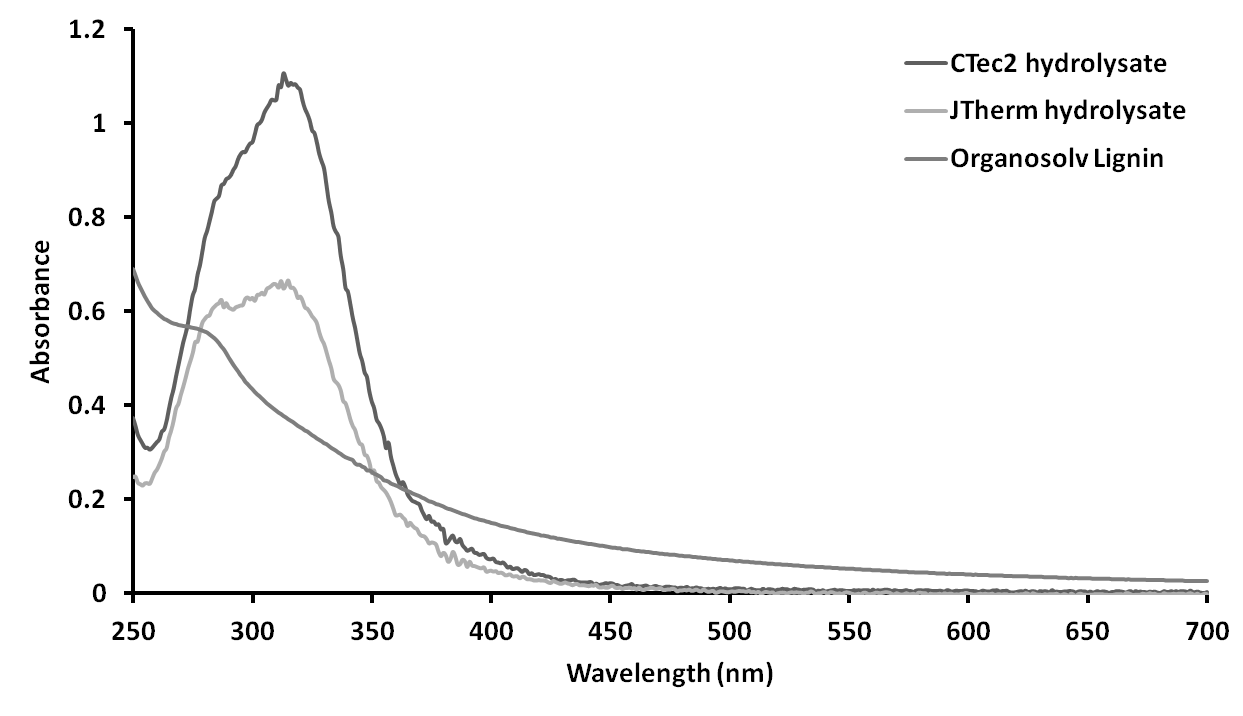


**Figure S2. UV/Vis absorbance scans of the Hydrolysates.** JTherm (from Figure 6) and CTec2 (from Figure 8) IL-pretreated switchgrass hydrolysates were diluted 20 fold and the water soluble fraction of organosolv lignin (Sigma #371033) was at 5 mg/ml. The hydrolysates and lignin have an absorbance peak at around 280 nm, and the hydrolysates have a second absorbance peak at around 315 nm. The CTec2 hydrolysate has a greater overall absorbance than JTherm, indicating higher concentrations of biomass-derived compounds are present in the CTec2 hydrolysate.
