# Supplementary material for: A Thermophilic Ionic Liquid-Tolerant Cellulase Cocktail for the Production of Cellulosic Biofuels
Source: PLoS One. 2012 May 23;7(5):e37010. doi: 10.1371/journal.pone.0037010 (PMC3359315; doi:10.1371/journal.pone.0037010)
Supplement: Figure S3 — Dose curve of the CTec2 cellulase cocktail on ionic-liquid pretreated switchgrass in M9 salts pH 5.0 at 50°C. The CTec2 dose is reported mg of enzyme product added per gram of total solids based on triplicate samples. (DOC) [file pone.0037010.s003.doc]

**
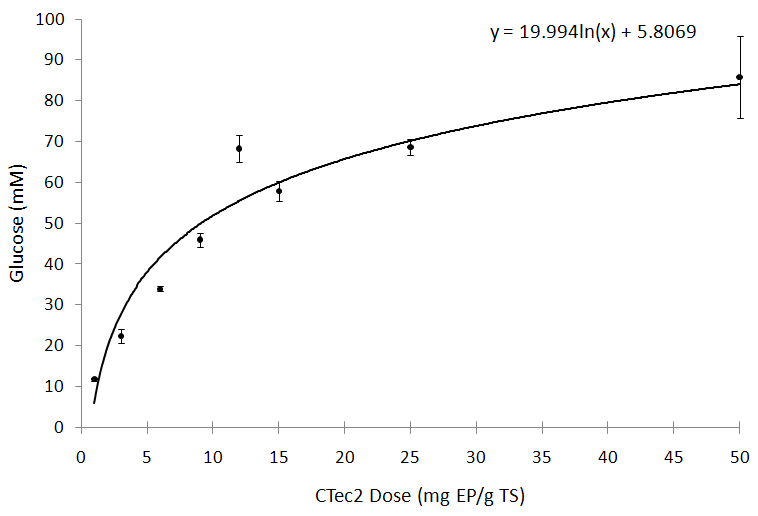
**

**Figure S3. Dose curve of the CTec2 cellulase cocktail on ionic-liquid pretreated switchgrass in M9 salts pH 5.0 at 50°C.** The CTec2 dose is reported mg of enzyme product added per gram of total solids based on triplicate samples.
