# Supplementary material for: A Thermophilic Ionic Liquid-Tolerant Cellulase Cocktail for the Production of Cellulosic Biofuels
Source: PLoS One. 2012 May 23;7(5):e37010. doi: 10.1371/journal.pone.0037010 (PMC3359315; doi:10.1371/journal.pone.0037010)
Supplement: Figure S4 — Growth of an E. coli strain engineered to produce biodiesel on CTec2 hydrolysate (A) and control samples containing 2% glucose and 1% xylose (B), 2% glucose and 1% xylose with the CTec2 enzyme product (C), and 2% glucose (D). Oxygen transfer rate (OTR), cell density (OD600), and sugar concentration were monitored during the fermentation to determine the impacts of the hydrolysate on growth and respiration. (DOC) [file pone.0037010.s004.doc]

**Figure S4. Growth of an *E. coli* strain engineered to produce biodiesel on CTec2 hydrolysate** (A) and control samples containing 2% glucose and 1% xylose (B), 2% glucose and 1% xylose with the CTec2 enzyme product (C), and 2% glucose (D). Oxygen transfer rate (OTR), cell density (OD_600_), and sugar concentration were monitored during the fermentation to determine the impacts of the hydrolysate on growth and respiration.
